# Photosynthetic conversion of carbon dioxide from cement production to microalgae biomass

**DOI:** 10.1007/s00253-023-12769-w

**Published:** 2023-09-21

**Authors:** Kathryn E. Dickinson, Kevin Stemmler, Tessa Bermarija, Sean M. Tibbetts, Scott P. MacQuarrie, Shabana Bhatti, Catherine Kozera, Stephen J.B. O’Leary, Patrick J. McGinn

**Affiliations:** Aquatic and Crop Resource Development, National Research Council of Canada, 1411 Oxford St., Halifax, N.S. B3H 3Z1 Canada

**Keywords:** Algae, Flue gas, Cement, *Chlorella*, Carbon capture, Emissions

## Abstract

**Abstract:**

Production of microalgae is a potential technology for capturing and recycling carbon dioxide from cement kiln emissions. In this study, a process of selecting a suitable strain that would effectively utilize carbon dioxide and generate biomass was investigated. A down-selection screening method was applied to 28 strains isolated from the area surrounding a commercial cement plant. In laboratory-scale (1 L) continuous-mode chemostats, observed productivity was > 0.9 g L^−1^ d^−1^ for most strains studied. *Chlorella sorokiniana* (strain SMC-14M) appeared to be the most tolerant to cement kiln gas emissions in situ, delivered under control of a pH-stat system, and was down-selected to further investigate growth and biomass production at large-scale (1000 L) cultivation. Results demonstrated little variability in lipid, crude protein, and carbohydrate composition throughout growth between kiln-gas grown algal biomass and biomass produced with laboratory grade CO_2_. The growth rate at which the maximum quantity of CO_2_ from the emissions is recycled also produced the maximum amount of the targeted biomass components to increase commercial value of the biomass. An accumulation of some heavy metals throughout its growth demonstrates the necessity to monitor the biomass cultivated with industrial flue gases and to carefully consider the potential applications for this biomass; despite its other attractive nutritional properties.

**Key points:**

• *Studied high biomass producing algal strains grown on CO*_*2*_ *from cement flue gas.*

• *Chlorella sorokiniana SMC-14M grew well at large scale, in situ on cement flue gas.*

• *Demonstrated the resulting commercial potential of the cultured algal biomass.*

## Introduction

Approximately 8% of industrial CO_2_ emissions responsible for global warming and consequent climate change arise from the production of cement, and in Canada, cement production contributes ~ 1.5 % of CO_2_ emissions (Gregg et al. 2008; CAC 2021). The emissions from these sources have been extensively studied, and the general scientific consensus is that they are, at least partially, responsible for our warming planet and associated climate change (Ellis et al. 2020; Mahlia 2002; Zhang et al. 2012). Rapid growth in developing economies, particularly in Asia, have placed a large demand on concrete and cement production. For every tonne of cement produced, approximately 900 kg of CO_2_ is generated (Benhelal et al. 2013; Hasanbeigi et al. 2010). Cement production is anticipated to reach 3.7–4.4 billion tonnes by 2050 due to the ever-increasing demand for concrete globally as a construction material (Hasanbeigi et al. 2010).

During cement manufacture, CO_2_ is typically released by three different processes. A significant portion of CO_2_ release (~40%) occurs during burning of fossil fuels in the pyro-processing unit which often employs the use of a calciner and kiln. About 10% can be attributed to transportation and electrical demands, and roughly 50% is released during the decomposition of CaCO_3_ and MgCO_3_ to produce CaO and MgO (Benhelal et al. 2013).

The negative ramifications of excessive CO_2_ release into our atmosphere has been identified as a key issue within the Kyoto Protocol in which industrialized countries and some European communities pledged to reduce their greenhouse gas emissions by 5% below 1990 levels within the 2008–2012 time frame (Balcilar et al. 2018). The reality is that, despite the agreements that were in place, emissions from 2000 to 2010 increased at a quicker rate than the previous three decades (Boden et al. 2011). A significant portion of those rising CO_2_ emissions can be linked to increased cement production in regions of growing population and economic activity, as China alone emits over 600,000 kilotonnes of CO_2_ through cement manufacturing while the second closest nation in terms of emissions is held by India at roughly 88,000 kilotonnes of CO_2_ (Padilla-Gamiño et al. 2013).

Despite efforts to reduce CO_2_ emissions, little can be done regarding the decomposition process of CaCO_3_ and MgCO_3_ to produce CaO and MgO (50% of emissions linked to cement production). As a result, this waste product is released into the atmosphere as flue gas. Since cement production cannot be decoupled from greenhouse gas production, significant R&D efforts are focused on the development of technologies that can intercept the resulting CO_2_ emissions before they dispersed into the atmosphere where they will accumulate. One such carbon capture technology under investigation is the integration of large-scale photosynthetic microalgae production directly linked to industrial emissions. Microalgae require CO_2_, a light source, as well as nutrients to convert CO_2_ into lipids, proteins, and carbohydrates (Pavlik et al. 2017). Many microalgal species such as *Chlorella* sp., *Chlamydamonas reinhardtii*, *Tetradesmus* sp. (formerly *Scenedesmus* sp.), *Nannochloropsis* sp., and *Nannochloris* sp. have been shown to sequester CO_2_ from flue gases (Ho et al. 2011). An added benefit of using microalgal carbon capture technologies is that it can be a revenue-generating activity through the harvesting of high-value compounds from the algae such as natural pigments (Cuellar-Bermudez et al. 2015) or use of the bulk biomass as a renewable alternative to fertilizers, a feedstock for the production of biobased materials and biofuels, or directly as food/feed.

Many cement producers are being proactive and are preparing for the future where carbon emissions will be constrained. In the cement industry, the effects of carbon pricing can be very high and it has been realized that the interception and recycling of CO_2_ emissions by microalgae would not only allow for more sustainable production of cement, but would also create a revenue stream in the form of the algal biomass itself to offset the costs of this CO_2_ mitigation strategy. However, there is some concern that microalgae cultivated in the presence of flue gas as the CO_2_ source may accumulate inhibitory toxic pollutants that would limit the uses of the harvested biomass.

The composition of flue gas will vary largely by location, but on average the composition typically ranges from 9.5 to 16.5% (v/v) CO_2_, 2–6.5% (v/v) O_2_, 100–300 ppm NO_x_, 280–320 ppm SO_x_, CO, heavy metals, and particulate matter (Lee et al. 2000). CO_2_ concentrations in flue gas above 10% and the presence of elevated levels of NO_x_ and SO_x_ are often toxic to microalgae (Cheng et al. 2019). It is therefore required to carefully select strains of microalgae that can tolerate the presence of these toxic pollutants while maintaining robust growth rates for the application of CO_2_ removal from flue gases (Cheng et al. 2019).

The aim of the current study was to evaluate the growth of selected strains of microalgae on CO_2_ emitted from the St. Marys Cement Inc. (Canada) (SMC) plant in St. Marys, Ontario, Canada, in order to evaluate the potential for this technology to convert waste CO_2_ into carbon-rich, value-added biomass through the process of photosynthesis. Within the kiln at the St. Marys cement plant, natural gas fires the production of clinker, an essential step in cement production. Combustion of natural gas contributes roughly 40% toward the cement plant’s overall carbon footprint. The remaining 60% of CO_2_ emissions originate from the limestone through calcination. Pond Technologies Inc. (PT), in collaboration with the National Research Council of Canada (NRC), co-located a microalgae cultivation and processing facility that allowed flue gas to flow to NRC “Brite-Box” photobioreactors (PBRs). The CO_2_-rich exhaust was redirected from the kiln at St. Marys though a 15-cm-thick insulated pipe to the facility. We designated this facility with the acronym SNP-MaPP to recognize the participation of the three primary stakeholders — SMC, NRC, and Pond Technologies followed by microalgae pilot plant. Local strains of microalgae were isolated from the grounds of the cement plant and the immediate surrounding area and screened for basic physiological traits. This included specific growth rate and biomass production potential as well as for tolerances to high CO_2_ and noxious flue gas constituents such as NOx emitted from the kiln. Algal biomass cultivated in two 1000 L PBRs using kiln gas was harvested, dried, and subjected to a comprehensive biochemical analysis in order to determine the particular potential application for which the biomass may be best suited, such as aquaculture feeds, fertilizers and/or soil conditioners, and bioplastics.

## Materials and methods

### Microalgae strain isolation and phylogenetic analysis

Water samples were collected from ponds adjacent to SMC’s cement plant (Votorantim Cimentos) and other bodies of water in and around the town of St. Marys, Ontario, Canada. A 10-μm plankton net (Aquatic Research Instruments, ID, USA) was suspended in the water column nearshore for several minutes and then drained to concentrate captured particles. Small samples were also obtained from biofilms adhered to submerged substrates and from sediments and humic debris near shore. These samples were vigorously homogenized in 500–1000 mL of sample water, followed by filtration through a stacked sieve consisting of three coupled 11.5 cm diameter polyvinyl chloride (PVC) pipe sections. The upper, middle, and lower sieves had an immobilized nitex mesh of 153 μm, 56 μm, and 33 μm, respectively. The 33 μm sieve filtrate was collected and divided into two, 40 mL aliquots and placed into 50 mL conical tubes for transport. Samples were placed into an illuminated cooler with ice at 4°C and transported back to the laboratory for strain isolation. Environmental and physical data, including water temperature (°C), salinity (ppt), total dissolved solids (TDS), pH, electrical conductivity (EC), dissolved oxygen (DO), and GPS coordinates, were recorded for all sampling sites. In the laboratory, field samples were enriched with growth media (*f*/2 or M8) in multi-well plates covered with Breathe-Easy sealing membranes (Sigma-Aldrich, St. Louis, MO, USA) and placed in transparent sealable containers modified with inlets and outlets for introducing gases. The transparent containers were incubated in a growth chamber (Conviron Controlled Environments, Winnipeg, MB, Canada) at 22°C under a continuous irradiance of 100 μmol photons m^−2^ s^−1^ and were flushed with air enriched to 3% CO_2_ for 1 h daily. After 1–2 weeks, wells with visible microalgal growth were transferred onto solidified agar plates (3% w/v agar) containing either *f*/2 or M8 media. A standard serial plating technique was used for microalgae isolation. Colonies were selected and transferred to new agar plates, and after several rounds of serial agar plating, single colonies were picked and transferred to liquid growth media. In total, 28 different isolates were recovered from colonies and cultured in their respective enrichment media. Strains isolated from St. Marys Cement (SMC) plant were assigned an identification number and designated with the letter “F” or “M” corresponding to the *f*/2 or M8 enrichment media, respectively, used during isolation. Strains were identified to the genus or species level by performing 18S and associated ITS sequence analysis of the ribosomal DNA obtained from unialgal cultures as described by Park et al. (Park et al. 2012). Standard techniques were used to conduct DNA extraction, PCR, and cloning. Extracted from transformed *Escherichia coli*, cloned plasmids underwent DNA sequencing at the Eurofins Genomics DNA sequencing laboratory. This sequencing utilized both forward and reverse primers provided with the cloning kit. Analysis of the obtained sequence data and comparison to related sequences accessed from the NCBI database revealed taxonomic assignments that exhibited ambiguity at the species level in some cases. Due to this uncertainty, we opted for a conservative approach in strain identification. Taxonomic designations were categorized cautiously at the genus level in some cases to ensure the accuracy of our reported findings among species branches that are currently poorly resolved. The 18S rRNA gene and ITS region sequences generated from this study were submitted to the National Center for Biotechnology (NCBI) GenBank database under accession numbers listed in Table 1. Any of the algal strains used in this study can be obtained from the National Research Council of Canada’s microalgae strain collection under a non-commercial research and development license upon written request to the corresponding author. From the 28 identified isolates (including 2 mixed cultures of 2 strains each), 14 were selected for initial screening in 1 L continuous culture vessels based on growth observations in flasks.

**Table 1:** Taxonomic classification, strain information, and NCBI accession numbers for 18S and ITS sequences of the microalgae strains isolated from the grounds of the St. Marys cement plant

Taxonomic designation	Strain Designation	18S	ITS
*Chlorella sorokiniana*	SMC-8M	OR393061	OR393080
	SMC-9M	OR393062	OR393081
	SMC-13M	OR393066	OR393085
	SMC-14M	OR393067	OR393086
	SMC-15M	OR393068	OR393087
	SMC-17M	OR393069	OR393088
	SMC-14F	OR393074	NA
*Chlorella* sp.	SMC-2M	OR393056	OR393075
	SMC-11M	OR393064	OR393083
	SMC-12M	OR393065	OR393084
	SMC-4F	OR393071	OR393090
	SMC-5F	OR393072	OR393091
	SMC-10F	NA	OR393096
	SMC-12F	OR393073	NA
*Desmodesmus* sp.	SMC-3M	OR393057	OR393076
	SMC-3F	OR393070	OR393089
*Tetradesmus obliquus*	SMC-5M	OR393059	OR393078
	SMC-6M	OR393060	OR393079
	SMC-6F	NA	OR393092
	SMC-7F	NA	OR393093
	SMC-8F	NA	OR393094
	SMC-15F	NA	OR393098
	SMC-16F	NA	OR393099
*Tetradesmus* sp.	SMC-4M	OR393058	OR393077
	SMC-10M	OR393063	OR393082
	SMC-9F	NA	OR393095
	SMC-13F	NA	OR393097
*Chlorella* sp./*Tetradesmus obliquus* mixed	SMC-16M	NA	NA

^a^SMC-16M was a mixed culture of *Tetradesmus obliquus* and *Chlorella* sp.

### Preliminary screening of selected microalgae strains in 1 L continuous culture systems

Cultivation of the 14 isolates (SMC-2M, 4M, 6M, 8M, 9M, 12M, 13M, 14M, 15M, 16M, 5F, 6F, 10F, and 14F) was conducted in 1 L glass vessels (Ace Glass Inc., Vineland, NJ, USA) fitted with water-jackets to maintain the cultivation temperature at 24°C. Culture vessels containing lake water were fitted with pre-calibrated pH electrodes, with inflow and outflow tubing and sterilized by autoclave when fully assembled. Continuous light was provided by strips of white LEDs wrapped uniformly around the water jacket. The irradiance rate was established by taking the mean of 9 transect points within the water-filled vessel using a PAR sensor (QSL-2100, Biospherical Instruments Inc., San Diego, CA, USA). Irradiance was increased during the cultivation period from 250 to 1000 μmol photons m^-2^ s^−1^. The pH level was maintained at 7.0 (± 0.05) through on-demand injection of CO_2_ through an automated pH-stat system. Aeration with filter-sterilized humidified air was introduced at a flow rate of 0.1 L min^−1^ and cultures were mixed with a magnetic stir bar at 350 rpm. Culture growth was initiated in batch mode with an initial inoculum of 2 × 10^6^ cells mL^−1^ in modified *f*/2 medium (5 × *f/2* nutrients and vitamins, and 2 × trace metals) and allowed to proceed until the culture reached stationary phase. Specific growth rate in exponential phase was estimated from the slope of the linear regression of the natural-log transformed cell numbers as follows:

1 $$\mu\ \left({d}^{-1}\right)=\mathit{\ln}\ \left({N}_2-{N}_1\right)/\left({t}_2-{t}_1\right)$$

After reaching stationary phase (24 h with no change in growth), samples were collected for biomass dry weights and continuous cultivation was initiated. Fresh growth medium was introduced into the culture vessel at a dilution rate of 0.6 d^−1^ and the culture outflow was removed at the same rate using a double pump-head using the same Masterflex peristaltic pump. In continuous mode at steady-state, the dilution rate is equivalent to the specific growth rate of the culture (ref).

2 $$\mu \left({d}^{-1}\right)=D\left({d}^{-1}\right)$$

The flow rate (F) (inflow of fresh medium and outflow of culture) is calculated based on the dilution rate and the volume (V) of the culture in the vessel:

3 $$F\left(L{d}^{-1}\right)=D\ \left({d}^{-1}\right)\times V(L)$$

**Fig. 1:**
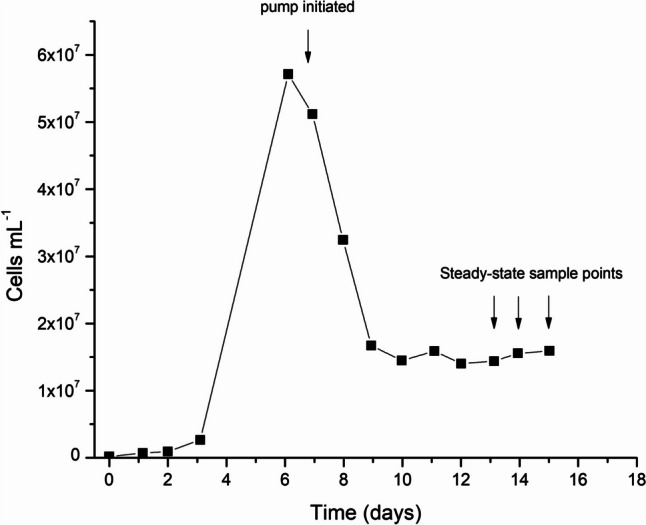
Culture growth (in this case, SMC-12M) was initiated in batch mode and allowed to proceed until stationary phase was reached. Peristaltic pumps were then initiated at a continuous dilution rate of 0.6 d^−1^. Cultures were considered to be at steady-state if the measured biomass concentration varied by less than 10% for at least 3 consecutive days. Once steady-state was achieved, samples were again collected for biomass dry weights

Cultures were considered to be at steady-state if the measured biomass concentration varied by less than 10% for at least 3 consecutive days. Once steady-state was achieved, samples were again collected for biomass dry weights (Fig. 1).

Algal cell concentrations were monitored daily using a Beckman-Coulter Multisizer 3 particle enumerator. Dry weights were determined gravimetrically by filtration onto pre-combusted 25 mm Whatman GF/F filters as previously described elsewhere (Dickinson et al. 2013).

### Secondary screening of selected microalgae strains in 10 L bioreactors at SMC/PT

Following the continuous cultivation trials at the NRC labs, nine microalgae strains (SMC- 2M, 6M, 12M, 13M, 14M, 15M, 5F, 6F, 14F) were screened for growth rate and biomass production in small photobioreactors at the algal production facility co-located at the cement plant. A secondary objective was to test the overall sensitivity of the algae to cement kiln emissions. Eighteen 20 L plastic buckets were top-fitted with a 50 W LED chip (640 nm peak emission) controlled by portable power supply units (Yescom DCP3010D) which provided light to drive photosynthesis and growth. Two flexible airlines fitted with 30 μm filters were inserted into the buckets for gas and air delivery. Pure kiln gas was redirected from the flue stack at St. Marys through a 15-cm-thick insulated pipe to a skid-mounted gas handling unit outside the facility. From here, flue gas was delivered to a bank of solenoid valves via a vacuum pressure pump (Model # 400-1941, Cole-Palmer), and air was provided using two air pumps (Model# ACO-9720, Hailea, Guangdong, China). Solenoids switched between delivering flue gas or air to each culture in response to a pH signal. At pH 7.2, pure kiln gas containing approximately 10% (v/v) CO_2_ was added to the cultures through a solenoid until the pH reached 6.8 at which time the flow of kiln gas was discontinued and replaced with air. The pH was monitored with a pH probe (Model# PHE-1311, Omega, Norwalk, CT, USA). The control of gas delivery via pH was through LabVIEW software. Ten liters of filter-sterilized, dechlorinated city water were added to the reactors and inoculated with 1 × 10^6^ cells mL^−1^ from cultures pre-cultivated in the same media. At the time of inoculation, cultures received a filter-sterilized dose of macronutrients and *f*/2 trace elements and vitamins. Every second day thereafter until the end of the cultivation period, the cultures received a dose of macronutrients only. Initial light intensity was adjusted to 250 μmol photons m^−2^ s^−1^ at the time of inoculation, 500 μmol photons m^−2^ s^−1^ between days 2 and 4, and 1000 μmol photons m^−2^ s^−1^ after day 5. Temperature was not controlled but stayed between 21 and 25°C. Trials lasted between 10 and 14 days, and samples were taken daily for cell enumeration using a particle analyzer (ZII, Beckman Coulter). Biomass samples were taken in duplicate on day 7 and on the final day of the trial, using the same protocol as with the 1 L experiments (Dickinson et al. 2013).

### Growth and biofixation of CO_2_ by selected microalgae strains in 1000 L Brite-Box™ photobioreactors at the SNP-MaPP facility

The down-selected microalgae strain (*Chlorella sorokiniana* SMC-14M) was then cultivated in 1000 L Brite-Box photobioreactors (PBRs) located at the SNP-MaPP facility to examine the effects of cement flue gas at this scale in situ on growth rate, biomass concentration, and on biochemical composition. Brite-Box PBRs are nominally 1000 L in volume and comprised of an enclosed fiberglass shell internally illuminated with 40, 32W fluorescent bulbs, arranged from top to bottom in five rows of eight bulbs per row. For more detailed information and a diagram of the Brite-Boxes, please refer to: Photobioreactor, Canadian patent number 2394518, 2012-05-22 available at Patent 2394518 Summary - Canadian Patents Database (https://www.ic.gc.ca/opic-cipo/cpd/eng/patent/2394518/summary.html). Duplicate Brite-Boxes were filled with city water and sterilized by the addition of 20 ppm of sodium hypochlorite which was allowed to mix in the PBRs for 24 h. Residual chlorine was neutralized through the addition of sodium thiosulphate pre-dissolved in one liter of deionized water. Three T-shaped PVC pipes suspended 12 cm from the floor of the PBRs provided vigorous mixing and aeration of air and gas delivered at 10 L min^−1^. The flue gas emissions contained in the kiln emissions stream were not constant; however, the flue gas was measured to be ~ CO_2_ 8-11 v/v%, O_2_ 18-20 v/v%, NO 11-92 ppm, CO 121-886 ppm, SO_2_ 9-20.5 ppm, and NO_2_ 11-92 ppm. The 1:1 mixture with air was injected into the PBRs under control of a pH stat system at pH 7.0±0.2, similar to the 10 L PBRs described in the previous section. As a control, 100% CO_2_ was blended with air to a final concentration of approximately 10% (*v/v*) provided to the cultures at a flow rate of 20 L min^−1^. The temperature of the cultures was maintained at 25°C through the use of a titanium cooling loop. The PBR cultures were fertilized with modified *f*/2 medium, and carboy-grown 20 L dense cultures were added as an initial inoculum by positive pressure displacement through the use of an air pump and filters. The light intensity at the time of inoculation was 720 μmol m^−2^ s^−1^ and increased to 960 and 1200 μmol m^−2^ s^−1^ on the second and third days, respectively.

Culture samples were obtained from a sampling port surface-sterilized with 70% (v/v) isopropyl alcohol and flushed to reduce sampling errors associated with settling. Approximately 10–15 mL of culture were taken for analysis. Sampling occurred twice a day to monitor the health of the culture, to obtain algal cell counts, and to obtain dry weights. Algal cell numbers were estimated with a Coulter counter (ZII, Beckman-Coulter, Pasadena, CA, USA). Cell culture health was assessed visually using an EVOS XL Core inverted brightfield microscope. The dry weights of the cultures were obtained according to the method of Dickinson et al. (Dickinson et al. 2013).

Larger samples of algal biomass for biochemical constituent analysis were obtained by harvesting portions of the algal culture through a small process centrifuge (Sharples) on cultivation days 2, 5, 7, and 9. Briefly, 18–20 L of culture was peristaltically pumped into the centrifuge at a feed rate of 1.5–3.0 L min^−1^ spinning at a rate of 1000 rpm. Wet algal paste was recovered, weighed, and frozen at −20°C until processed further.

### Analysis of microalgal biomass

Frozen algal pastes were lyophilized for 72 h at a low shelf temperature (≤5°C) in a freeze-dryer (model 35EL, The Virtis Company, Gardiner, NY), finely ground by hand using a mortar and pestle and stored at −80°C [15]. Moisture and ash contents were determined gravimetrically by drying in an oven at 105°C and by incineration in a muffle furnace at 550°C for 18 h. Nitrogen (N) contents were determined by elemental analysis (950°C furnace) using a Leco N analyzer (model FP-528, Leco Corporation, St. Joseph, MI) with ultra-high purity oxygen as the combustion gas and ultra-high purity helium as the carrier gas. Crude protein contents of SMC-14M *C. sorokiniana* samples were calculated using the conventional nitrogen-to-protein (N-to-P) conversion factor (N×6.25) and a generalized microalgae N-to-P conversion factor (N×4.78; Lourenço et al. 2004). Lipids were extracted by methanolic HCl in situ transesterification (McGinn et al. 2012), and the corresponding fatty acid methyl esters (FAMEs) were separated and quantified by GC-FID (Omegawax 250 column, Agilent 7890). Individual FAs, along with an internal standard (C19:0; methyl nonadecanoate, Fluka), were identified by comparing retention times to two FA reference mixtures (Supelco 37 and PUFA No. 3, Sigma-Aldrich). Carbohydrate contents were determined by colorimetry using phenol and sulfuric acid following acid hydrolysis (2.5 M HCl at 95°C for 3 h) (Dubois et al. 1956; Sukenik et al. 1993). Final results were determined against a dextrose standard curve (0–100 μg mL^−1^; D-glucose, solid, >99% pure, Sigma-Aldrich, Cat. G5400). Gross energy (MJ kg^−1^) contents were measured using an isoperibol oxygen bomb calorimeter (model 6200, Parr Instrument Company, Moline, IL) calibrated using benzoic acid and equipped with a Parr 6510 water handling system for closed-loop operation. Amino acid concentrations were determined using the Waters Pico-Tag RP-HPLC method (Heinrikson and Meredith 1984), and the essential amino acid index (EAAI) was calculated according to (Oser 1951) relative to an ideal protein pattern (egg albumin). Elemental compositions were measured by ICP-AES according to SW-846 Method 6010C, and mercury was measured following reference method 7471B (US EPA 2007). Concentrations of minerals, trace elements, and heavy metals were determined using element-specific wavelengths on an IRIS Intrepid II spectrometer (Thermo Fisher Scientific, Waltham, MA). All analytical work was conducted in triplicate.

### Statistical methods

Data are reported as mean ± standard deviation. Statistical analyses were performed using one-way repeated measured analysis of variance (RM-ANOVA) (SigmaStat® v.3.5) with a 5% level of probability (*P*<0.05) selected in advance to sufficiently demonstrate a statistically significant difference. Where significant differences were observed, treatment means were differentiated using pairwise comparisons using the Tukey test. Raw data was checked for normality using the Kolmogorov-Smirnov test (SigmaStat® v.3.5).

## Results

The various taxa of microalgae isolated from ponds adjacent to the SNP-MaPP and surrounding areas in the town of St. Marys are listed in Table 1. Twenty-nine isolates were obtained by enriching the sample cultures with either M8 or *f*/2 medium. These isolates were identified to either the genus or species level by performing 18S and ITS sequence analysis on genes coding for rDNA. Microalgae from three genera were isolated, including *Chlorella*, *Tetradesmus* (formerly *Scenedesmus*), and *Desmodesmus*. From the 28 isolates, 14 were chosen to examine in 1 L continuous chemostats based on growth observations in flasks (data not shown).

**Fig. 2:**
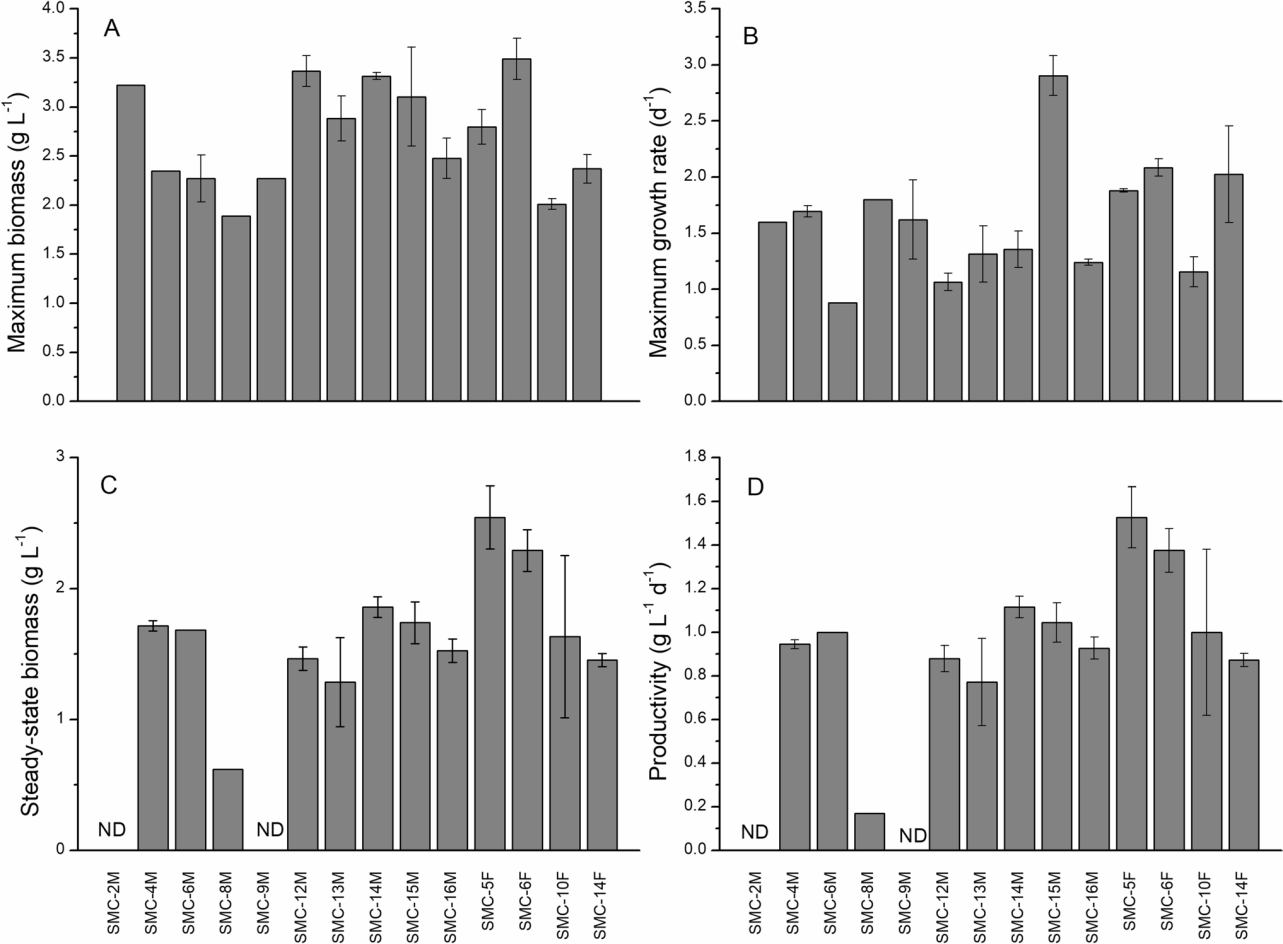
**A** Biomass yield (g L^−1^) and **B** maximum growth rate (d^−1^) for 14 strains cultivated in 1 L vessels in batch mode. Data represent means (*n* = 2 ± SD). **C** Steady-state biomass production (g L^−1^) and **D** productivity (g L^−1^ d^−1^) for 14 strains in 1 L continuous chemostat culture at a dilution rate of 0.6 d^−1^. Data represent means (*n* = 6 ± SD)

Maximum growth rate and biomass yield were examined in batch cultures (Fig. 2A, C). The strains that had the greatest biomass at stationary phase were SMC-2M, SMC-12M, SMC-14M, SMC-15M, and SMC-6F, all with dry weights > 3 g L^−1^. The strain that achieved the most biomass was SMC-6F with 3.49 g L^−1^. The strains showing the fastest maximum specific growth rates were SMC-2M, SMC-4M, SMC-8M, SMC-15M, SMC-5F, SMC-6F, and SMC-14F with growth rates greater than 1.6 d^−1^. SMC-15M had the highest overall growth rate at 2.91 d^−1^.

Once the pumps were started and a continuous steady state was achieved and stable, it was observed that productivity was high for most strains (>0.9 g L^−1^d^−1^) (Fig. 2B, D). A constant daily biomass of 2.54 g L^−1^ was achieved by SMC-5F and 2.29 g L^−1^ for SMC-6F. The productivity of the strains (excluding SMC-8M, low; SMC-5F, high) averaged 0.99 ± 0.17 g L^−1^ d^−1^ at a steady dilution rate of 0.6 d^−1^. The robustness of certain strains to acclimate to the imposed conditions and to maintain continuous growth at productivities that were deemed acceptable, allowed for the advancement to the next stage of screening. Strains were eliminated at this stage for the following reasons: SMC-4M was productive, with a moderate maximum growth rate, although a lower maximum biomass. However, it had a tendency to form clumps and to stick to the walls of the glass culturing vessels; SMC-8M had a low maximum biomass and low productivity in continuous culture; SMC-9M did not survive once continuous pumps were started most likely due to bacterial contamination; SMC-16M was eliminated simply due to being a mixed culture, as it was decided that a unialgal culture was preferable for the next phase.

**Fig. 3:**
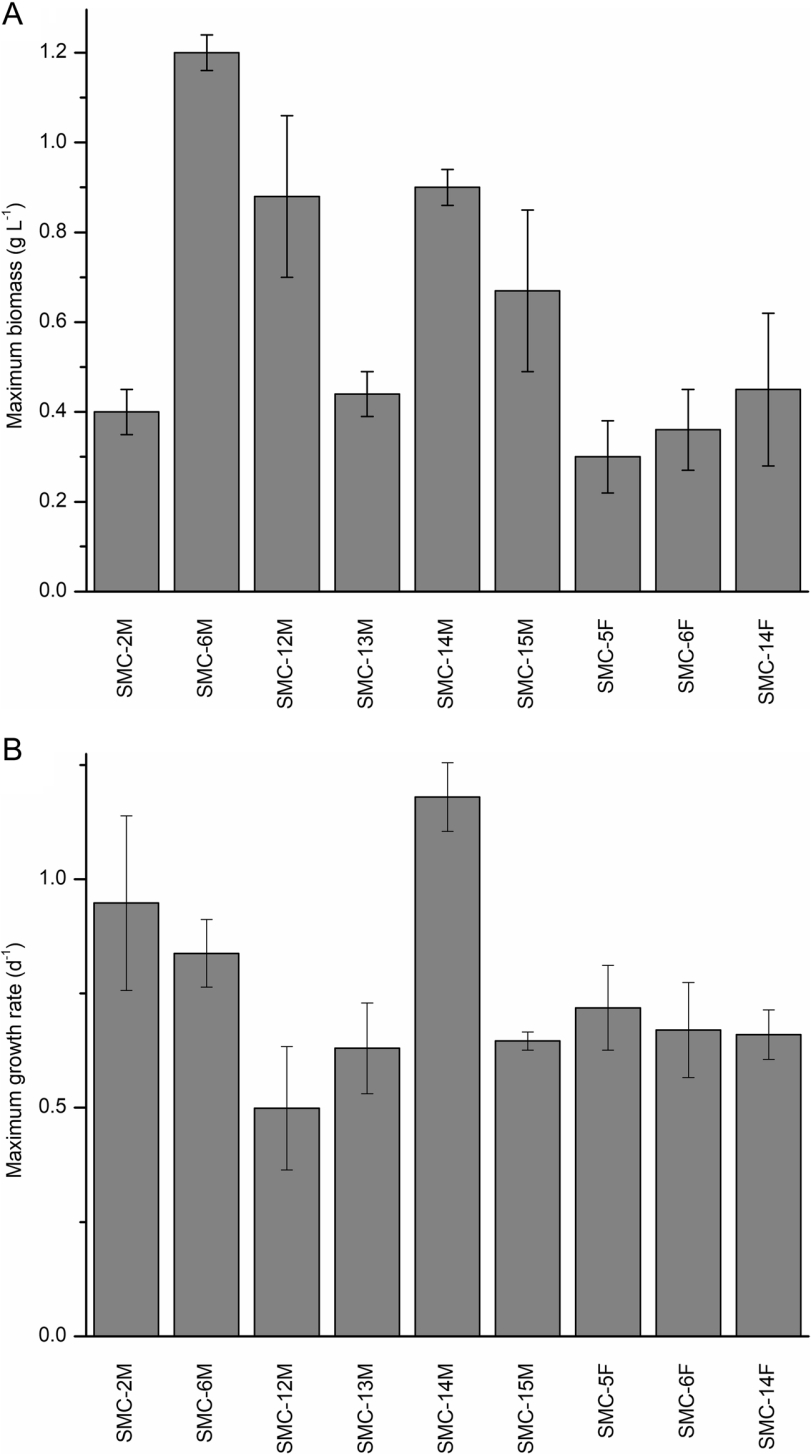
**A** Biomass yield (g L^−1^) and **B** maximum growth rate (d^−1^) of nine isolates down-selected for screening in 10 L bioreactors with cement kiln flue gas emissions. Data points represent means ± s.d. of two separate analytical determinations

Nine strains determined from the chemostat experiments were further examined in 10 L bucket bioreactors at the SNP-MaPP facility to evaluate their growth potential using actual flue gas as the carbon source (Fig. 3). Pure kiln flue gas containing ~ 8–11% CO_2_ was added through a pH stat system to maintain the pH between 6.8 and 7.2. It was observed that some strains were more tolerant to flue gas as a CO_2_ source than others. Under these conditions, SMC-6M had the highest maximum biomass of the strains tested with 1.2 g L^−1^, followed by SMC-14M and SMC-12M (Fig. 3A). For most strains, the maximum growth rate averaged 0.67±0.14 with the exception of SMC-14M which demonstrated a higher growth rate of 1.18 d^−1^ (Fig. 3B). SMC-14M, a strain identified as *Chlorella sorokiniana*, was selected to move on to larger scale growth experiments at 1000 L due to its tolerance to flue gas and ability to achieve comparably high maximum biomass at a high maximum growth rate.

Several trial runs were conducted in two 1000 L Brite-Box PBRs simultaneously to observe the growth potential of SMC-14M using cement flue gas emissions. Due to occasional kiln shutdowns inherent with commercial cement plants, there were periods of irregular flue gas supply. The gases contained in the kiln emissions stream were not constant; however, the flue gas was measured to be ~ CO_2_ 8-11 v/v%, O_2_ 18-20 v/v%, NO 11-92 ppm, CO 121-886 ppm, SO_2_ 9-20.5 ppm, and NO_2_ 11-92 ppm, and the 1:1 mixture with air was injected into the PBRs. Six trials of 2 replicates each were conducted, and results of maximum growth rate and maximum biomass are listed in Table 1. The average maximum growth rate of microalgae grown on flue gas was found to be 1.01±0.26 d^−1^, and the maximum biomass averaged 0.39±0.15 g L^−1^. Another trial was conducted with two replicate PBRs using commercially supplied CO_2_ blended with air. There were no significant differences in both maximum growth rate results (*P*=0.80), and biomass concentration (*P*=0.47) between the control and the flue gas trials (Table 2).

**Table 2:** Biomass yield (g L^−1^) and maximum growth rate (d^−1^) of SMC-14M (*Chlorella sorokiniana)* cultivated in 1000 L Brite-Box PBRs grown on CO_2_ and cement flue gas emissions. Data represent means of two separate determinations

Trial #	Specific growth rate (_d_^−1^)	Biomass yield (_g L_^−1^)
Control	0.74±0.29	0.35±0.13
Flue gas 1	1.10	0.39
Flue gas 2	0.84	0.65
Flue gas 3	0.83	0.57
Flue gas 4	0.96	0.34
Flue gas 5	1.24	0.40
Flue gas 6	0.90	0.17
Flue gas 7	0.99	0.42
Flue gas 8	1.56	0.24
Flue gas 9	0.67	0.36
Flue gas average	1.01±0.26	0.39±0.15

**Fig. 4:**
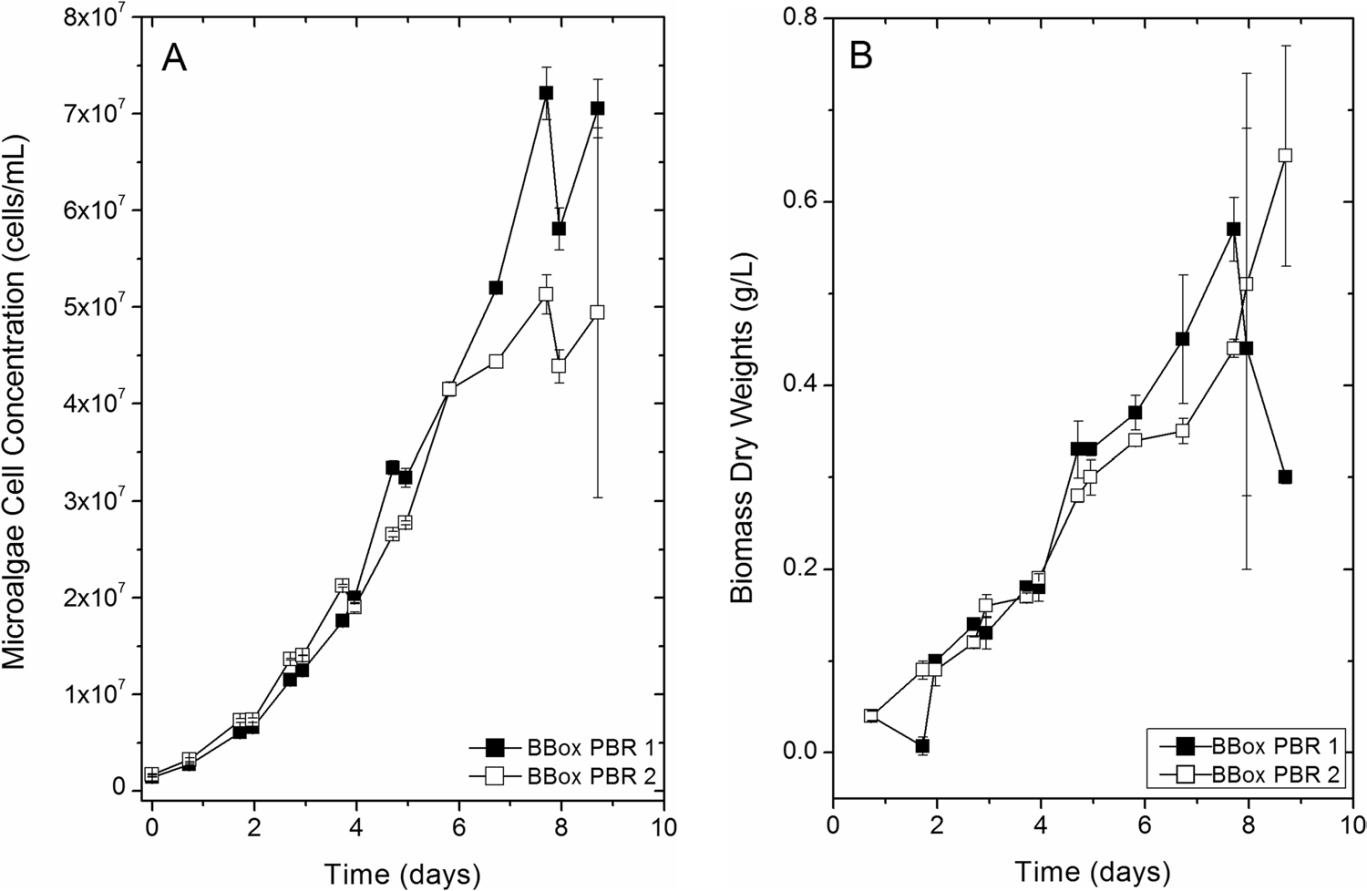
**A** Cell concentration (cells mL^−1^) and **B** biomass dry weight (g L^−1^) of SMC-14M (*Chlorella sorokiniana*) cultivated in 1000 L Brite-Boxes grown on CO_2_ from cement flue gas emissions. Data points represent mean ±SD of two separate analytical determinations

For one such trial, samples were taken at various stages of the batch mode growth cycle in order to examine the biochemical composition of the algal biomass as the culture aged (Fig. 4A, B; Tables 3, 4, 5, and 6).

**Table 3:** Proximate composition (dry weight basis) of SMC-14M (*Chlorella sorokiniana*) after 2, 5, 7, and 9 days of cultivation in 1000 L Brite-Box PBRs aerated with cement kiln flue gas at the St/Po/Nr pilot-scale facility (*n*=2). Values within the same row having different superscript letters are significantly different (*P*<0.05)

	Day 2	Day 5	Day 7	Day 9	*P-*value
Ash (%)	11.8±1.1^a^	10.9±2.3^ab^	9.0±0.8^bc^	8.8±0.7^c^	0.001
Crude protein (%N×6.25)	67.0±0.5^ns^	66.4±2.1	66.7±2.5	68.2±0.7	0.053
Crude protein (%N×4.78)^b^	51.2±0.4^ns^	50.8±1.6	51.0±1.9	52.2±0.5	0.053
Total FAME (%)	8.7±0.1^ns^	9.2±0.3	8.6±1.4	9.4±1.5	0.250
Carbohydrate (%)	14.3±0.4^ns^	14.7±0.1	16.8±4.4	15.1±3.4	0.754
Gross energy (MJ kg^−1^)	21.3±0.3^ns^	21.5±0.8	21.7±0.8	22.0±0.3	0.208

^a^Cultivations conditions were pH 6.4, 25°C with nutrients *f*/2 modified with 2x nitrate and phosphate, for 9 days and peak exponential growth rate was 0.72 d^−1^

^b^Lourenço et al. (2004)

**Table 4:** Essential amino-acid profile of SMC-14M (*Chlorella sorokiniana*) after 2, 5, 7, and 9 days of cultivation in 1000 L Brite-Box PBRs aerated with cement kiln flue gas at the SNP-MaPP pilot-scale facility (*n*=2). Data are expressed as g each amino-acid per 100 g protein (g 100 g protein). Values within the same row having different superscript letters are significantly different (*P* <0.05)

	Day 2	Day 5	Day 7	Day 9	*P-*value
Arginine	6.3±0.3^a^	5.7±0.1^ab^	5.9±0.1^ab^	5.5±0.2^b^	0.028
Histidine	1.9±0.2^ns^	2.0±0.0	1.7±0.1	2.0±0.1	0.140
Isoleucine	4.0±0.0^ns^	4.1±0.0	4.2±0.2	4.2±0.1	0.569
Leucine	8.8±0.1^ns^	8.5±0.1	8.7±0.1	8.7±0.0	0.199
Lysine	7.3±0.0^ns^	7.4±0.1	7.5±0.3	7.3±0.3	0.757
Methionine	0.0±0.0^b^	1.6±0.0^a^	0.3±0.4^b^	1.5±0.1^a^	0.007
Phenylalanine	5.1±0.0^ns^	5.0±0.0	5.2±0.0	5.2±0.1	0.060
Threonine	4.3±0.2^ns^	3.9±0.0	4.2±0.1	3.9±0.0	0.112
Tryptophan	2.6±0.1^a^	2.3±0.0^bc^	2.4±0.0^ab^	2.1±0.1^c^	0.007
Valine	6.8±0.5^ns^	6.8±0.1	6.9±0.1	6.8±0.1	0.925
EAAI	1.0±0.0^ns^	1.0±0.0	0.9±0.0	0.9±0.0	0.200

**Table 5:** Total esterifiable fatty acids profile of SMC-14M (*Chlorella sorokiniana*) after 2, 5, 7, and 9 days of cultivation in 1000 L Brite-Box PBRs aerated with cement kiln flue gas at the SNP-MaPP pilot-scale facility (*n*=2). Data for each fatty acid are expressed as a % of the total esterifiable fatty acid pool. Values within the same row having different superscript letters are significantly different (*P*<0.05)

	Day 2	Day 5	Day 7	Day 9	*P*-value
16:0	14.5±0.5^a^	11.5±0.8^b^	12.6±1.3^b^	12.3±1.2^b^	<0.001
16:1n-7	3.7±0.5^b^	5.3±0.9^a^	6.1±1.3^a^	5.6±0.8^a^	<0.001
16:2n-6	3.8±0.2^c^	6.8±0.3^b^	7.7±0.7^a^	8.3±0.1^a^	<0.001
17:1	24.5±0.4^a^	24.0±0.3^a^	21.3±2.5^b^	21.7±2.5^ab^	0.003
18:2n-6	10.9±1.7^b^	11.9±0.4^ab^	13.9±2.2^a^	13.9±2.1^a^	0.001
18:3n-3 (ALA)	42.5±1.5^a^	40.5±0.2^ab^	38.3±3.1^b^	38.2±1.8^b^	<0.001
Ʃ SFA	14.5±0.5^a^	11.5±0.8^b^	12.6±1.3^b^	12.3±1.2^b^	<0.001
Ʃ MUFA	28.2±1.0^ns^	29.3±1.2	27.4±1.2	27.3±1.6	0.061
Ʃ PUFA	57.3±0.4^c^	59.2±0.5^b^	60.0±0.2^a^	60.4±0.4^a^	<0.001
Ʃ n-3 PUFA	42.5±1.5^a^	40.5±0.2^ab^	38.3±3.1^b^	38.2±1.8^b^	<0.001
Ʃ n-6 PUFA	14.8±1.9^c^	18.7±0.7^b^	21.6±2.9^a^	22.2±2.2^a^	<0.001
n-3:n-6 ratio	2.9±0.5^a^	2.2±0.1^ab^	1.8±0.4^bc^	1.7±0.2^c^	<0.001

**Table 6:** Total mineral, trace element, and heavy metal profiles of SMC-14M (*Chlorella sorokiniana*) after 2, 5, 7, and 9 days of cultivation in 1000 L Brite-Box PBRs aerated with cement kiln flue gas at the SNP-MaPP pilot-scale facility (*n*=2). Data for each are expressed as a % of the total biomass (mg kg^−1^). Values within the same row having different superscript letters are significantly different (*P* <0.05)

	Day 2	Day 5	Day 7	Day 9	*P*-value
Minerals (%)					
Calcium	na	0.7±0.7^ns^	0.5±0.3	0.3±0.1	0.241
Magnesium	na	0.3±0.0^ns^	0.3±0.0	0.3±0.0	0.904
Phosphorous	na	1.7±0.4^ns^	1.5±0.1	1.3±0.1	0.285
Potassium	na	1.4±0.1^ns^	1.3±0.1	1.2±0.2	0.386
Sodium	na	0.1±0.0^ns^	0.1±0.0	0.1±0.0	0.108
Ca:P ratio	na	0.3±0.3^ns^	0.3±0.2	0.2±0.1	0.183
Trace elements (mg kg^−1^)					
Aluminum	na	57.0±9.7^a^	14.8±2.4^b^	6.5±1.7^b^	<0.001
Antimony	na	<DL	<DL	<DL	-
Barium	na	44.1±18.2^a^	20.4±7.0^b^	7.1±0.4^b^	0.007
Beryllium	na	<DL	<DL	<DL	-
Bismuth	na	<DL	<DL	<DL	-
Chromium	na	1.9±0.1^ns^	1.3±0.3	1.3±0.7	0.333
Cobalt	na	0.3±0.1^ns^	0.3±0.0	0.4±0.1	0.167
Copper	na	60.0±7.0^ns^	69.0±18.4	75.6±42.2	0.904
Iron	na	1186.9±58.7^a^	601.1±17.3^b^	368.6±26.3^c^	<0.001
Lithium	na	0.1±0.1^ns^	0.1±0.1	0.1±0.1	0.489
Manganese	na	110.5±54.0^ns^	113.3±21.9	88.0±15.8	0.183
Molybdenum	na	1.1±0.1^ns^	1.0±0.3	1.0±0.2	0.861
Nickel	na	0.5±0.1^ns^	0.6±0.1	0.4±0.1	0.244
Selenium	na	<DL	<DL	<DL	-
Silver	na	<DL	<DL	<DL	-
Strontium	na	481.5±464.0^ns^	367.0±261.1	185.7±79.9	0.141
Thallium	na	<DL	<DL	<DL	-
Titanium	na	1.2±0.4^a^	0.7±0.3^b^	0.4±0.1^b^	0.003
Vanadium	na	4.1±0.5^ns^	3.9±0.0	3.8±0.2	0.465
Zinc	na	47.5±2.6^ns^	43.6±4.5	39.7±4.7	0.244
Zirconium	na	0.3±0.1^ns^	0.2±0.0	0.2±0.1	0.108
Heavy metals — biomass (ppm)^a^					
Arsenic	na	<DL	<DL	<DL	-
Cadmium	na	0.3±0.2^ns^	0.5±0.4	0.6±0.4	0.179
Lead	na	<DL	<DL	0.4±0.5	-
Mercury	na	0.3±0.2^b^	0.8±0.7^ab^	0.9±0.7^a^	0.038
Heavy metals — supernatant (ppm)					
Arsenic (DL = 0.0001)	<DL	<DL	<DL	<DL	-
Cadmium (DL = 0.00001)	<DL	<DL	<DL	<DL	-
Lead (DL = 0.00001)	<DL	<DL	<DL	<DL	-
Mercury (DL = 0.00001)	<DL	<DL	<DL	<DL	-

^a^Maximum allowable concentration (ppm) in animal feed ingredients = arsenic (2–4), cadmium (0.5–1.0), lead (10–40), and mercury (0.1–0.4)

*ns* no significant difference

*< DL* below detection limit

*na* not analyzed

Biochemical composition of *Chlorella sorokiniana* (SMC-14M) cultivated in duplicate 1000 L Brite-Box PBRs at SMC/PT on kiln flue gas is shown in Table 3 (presented on a 100% dry weight basis). Four separate biomass samples were obtained in a time-course during the growth of SMC-14M in the PBRs — on days 2, 5, 7, and 9 (Fig. 4A, B). In terms of proximate composition, ash content significantly declined over the course of the cultivation period (11.8–8.8%; *P*=0.001). However, no significant differences were observed for crude protein (*P*=0.053), lipid (*P*=0.250), carbohydrate (*P*=0.754), or gross energy (*P*=0.208) as the cultures aged (Table 3). The composition of essential amino acids (g 100 g protein^−1^) differed significantly for arginine (5.5–6.3; *P*=0.028), methionine (0.0–1.6; *P*=0.007), and tryptophan (2.1–2.6; *P*=0.007) over the culture growth period, while levels were statistically the same for the other amino acids (*P*≥0.060) (Table 4). The majority of lipid (>85% of total FAs) in strain SMC-14M biomass was composed of 16:0, 17:1, 18:2n-6, and 18:3n-3. Fatty acids 16:0, 17:1, and 18:3n-3 all generally decreased over time with values of 14.5–11.5 (*P*<0.001), 24.5–21.3 (*P*=0.003), and 42.5–38.2 (*P*<0.001), respectively, while those of 18:2n-6 increased over time (10.9–13.9; *P*=0.001) (Table 5). Minor FAs that generally increased over time included 16:1n-7 (3.7–6.1; *P*<0.001) and 16:2n-6 (3.8–8.3; *P*<0.001). Total saturated fatty acids (Σ SFA) significantly decreased over time (14.5–11.5; *P*<0.001), total polyunsaturated fatty acids (Σ PUFA) significantly increased over time (57.3–60.4; *P*<0.001), and total monounsaturated fatty acids (Σ MUFA) remained statistically unchanged (27.3–29.3; *P*=0.061). The rising Σ PUFA was comprised of significantly increasing n-6 PUFA (14.8-22.2; *P*<0.001) and significantly decreasing n-3 PUFA (42.5–38.2; *P*<0.001), which resulted in a statistically significant decreasing n-3:n-6 ratio over the cultivation period (2.9–1.7; *P*<0.001) (Table 5). The compositions of major minerals (%) were statistically similar over cultivation time for calcium (0.3–0.7; *P*=0.241), magnesium (0.3; *P*=0.904), phosphorous (1.3–1.7; *P*=0.285), potassium (1.2–1.4; *P*=0.386), sodium (0.1; *P*=0.108), and the Ca:P ratio (0.2–0.3; *P*=0.183) (Table 6). Trace element compositions (mg kg^−1^) were statistically similar for chromium (1.3–1.9; *P*=0.333), cobalt (0.3–0.4; *P*=0.167), copper (60.0–75.6; *P*=0.904), lithium (0.1; *P*=0.489), manganese (88.0–113.3; *P*=0.183), molybdenum (1.0–1.1; *P*=0.861), nickel (0.4–0.6; *P*=0.244), strontium (185.7–481.5; *P*=0.141), vanadium (3.8–4.1; *P*=0.465), zinc (39.7–47.5; *P*=0.244), and zirconium (0.2–0.3; *P*=0.108) (Table 6). Significant decreases over time were observed for aluminum (57.0–6.5; *P*<0.001), barium (44.1–7.1; *P*=0.007), iron (1186.9–368.6; *P*<0.001), and titanium (1.2–0.4; *P*=0.003) (Table 6). Analyzed trace elements that fell below detection limits included antimony, beryllium, bismuth, selenium, silver, and thallium. Heavy metal concentrations (ppm) in the biomass were statistically similar for cadmium (0.3–0.6; *P*=0.179), while mercury levels increased over time (0.3–0.9; *P*=0.038), and arsenic and lead levels were generally below detection limits. Heavy metal concentrations (ppm) were also assayed for in the supernatant water collected at each harvest and those of arsenic, cadmium, lead, and mercury all fell below detection limits (Table 6).

## Discussion

Utilization of cement plant flue gas in situ for microalgal cultivation supports the sustainable recycling of CO_2_ emissions and represents a potentially more economical approach in a carbon-constrained future. Although there have been previous studies examining the use of industrial flue gas as a carbon source for algal growth (Chen et al. 2012; Cuellar-Bermudez et al. 2015; Jin et al. 2020; Lee et al. 2000; Yen et al. 2015), data collected from real-world studies using actual flue gas are limited. Only under these conditions can realistic challenges such as interruptions in CO_2_ supply and the presence of NO_x_ and SO_x_ gases be assessed for their impacts on algal biomass production and processing.

The aim of this study was to select a robust microalgal strain with tolerance to a high but also variable concentration of CO_2_ and noxious pollutants in the flue gas stream. The screening process involved first isolating native strains of algae from the grounds of the cement plant itself, bringing them into culture and evaluating their capacity for growth on actual flue gas streams on-site. The use of native strains in this study and for potential future commercial deployment was deemed to be environmentally responsible as it eliminated the potential of introducing exotic and potentially invasive, microalgae strains into the surrounding area. Growth rate and overall biomass accumulation (proportional to carbon fixation) were used as selection criteria to determine which isolates would be used for testing with flue gas from the cement plant. Other physical criteria important during down-selection included potential for biofilm formation, clumpiness and foaming, which were deemed undesirable. Strains which demonstrated one or more of these traits were set aside and not used for screening in the 1000L Brite-Box PBRs (Singh and Sharma 2012).

All down-selected strains were green algae belonging to the genera *Chlorella*, *Tetradesmus* (formerly *Scenedesmus*), and *Desmodesmus*. The initial screening in 1 L chemostats demonstrated that native isolates were able to achieve high growth rates and biomass productivity consistent with other green algae strains (Li et al. 2019; Seyfabadi et al. 2011). In continuous chemostat culture conditions, most strains performed well with steady-state dry weight yields of >1.4 g L^−1^ at a dilution rate of 0.6 d^−1^. This dilution rate of 0.6 d^−1^ was chosen based on previous studies (Dickinson et al. 2013) in which the biochemical composition and the productivity of culture could be “tuned” through adjustment of the culture dilution rate. In this particular system where carbon capture was the main goal, maximum biomass productivity using a minimum of resources was desired. With *Scenedesmus* sp. a dilution rate of 0.6 d^−1^ fit these requirements (Dickinson et al. 2013). Cho et al. (Dae-Hyun Cho et al. 2016) conducted a similar study examining a *Chlorella vulgaris* strain and dilution rates between 0 and 1.6 d^−1^. They found that the greatest biomass productivity was achieved at 0.75 d^−1^ with 1.01 g L^−1^ d^−1^. This rate of productivity and others reported for green algae (Ramos Tercero et al. 2014) is comparable to results observed here, which averaged 0.99 ± 0.17 g L^−1^d^−1^ and was therefore deemed appropriate for the next stage of screening.

Nine strains (made up of *Chlorella* and *Tetradesmus* strains) were down-selected showing growth and carbon capture characteristics suitable for cultivation in the conditions available at the SNP-MaPP plant. These strains were then assessed for sensitivity to the cement kiln emissions. Emissions containing 8–11% CO_2_ were delivered using a pH-stat system to maintain a pH of 7.0±0.2. This is a CO_2_ concentration that falls within the range of CO_2_ generally present in industry flue gases, which typically range from 3 to 15% CO_2_ (Packer 2009). Microalgae have varying levels of tolerance to high CO_2_ concentrations, and many studies have examined these limits (Dickinson et al. 2019; Jin et al. 2020). A comparison of microalgal strains (*Tetradesmus* (*Scenedesmus*), *Botryococcus*, and *Nannochloropsis* spp.) was presented by Cuellar-Bermudez et al. (Cuellar-Bermudez et al. 2015), demonstrating tolerance to CO_2_ concentrations up to 50%. Jin et al. (Jin et al. 2020) examined *Chlorella*, *Heynigia*, *Desmodesmus*, and *Scenedesmus* and found preferences for CO_2_ concentrations of up to 15% (*Heynigia*). Studies examining actual flue gas in situ are more limited, and even scarcer are studies examining the use of flue gas emissions at large scale; however, with financial incentives for lowering CO_2_ emissions in place in many jurisdictions, research is increasing in this area. Chen et al. (2012) report success with the cultivation of *Spirulina* in 15 L containers at a coal fired power plant in Taiwan. Their system involved stacking these smaller scale containers to maximize production. Yadav et al. (Yadav et al. 2015) examined the effects of flue gas from a thermal power station on a strain of *Chlorella* at bench scale and concluded there was some economical potential in recycling CO_2_ from flue gas emissions through algae. In addition, researchers (Heinrikson and Meredith 1984; Oser 1951; Jin et al. 2020; Chen et al. 2012) have demonstrated that *Chlorella* sp. can tolerate NO_x_ and SO_x_ compounds emitted from actual industrial flue gas sources without inhibition to growth. Our findings with *Chlorella sorokiniana* agree with these reports. In the present study, SMC-14M in 10 L photobioreactors demonstrated a higher tolerance to flue gas emissions than the other strains examined and had a high growth rate and maximum biomass production. This strain was therefore chosen for the larger scale (1000 L) studies.

It is well known that volumetric biomass productivity decreases with increasing bioreactor volume due to increasingly inefficient light distribution through the PBR. Microalgae strains have varying photosynthetic efficiencies with some more tolerant to changes in irradiance levels and light/dark cycling than others (Carvalho et al. 2011). We have shown previously (Dickinson et al. 2019) that light quality can affect the productivity of *Chlorella* sp. (formerly *Micractinium inermum*) (SMC-5F), and these effects could be exacerbated by high CO_2_ concentrations. In this study, although we expected a decrease in maximum biomass overall simply due to scaling up to a larger bioreactor, our aim was to examine any compounding effects of stress at this level due to the differences in CO_2_ sources. Nitrous oxide (NO) and nitrogen dioxide (NO_2_), the major NOx species in these gases, are environmental pollutants in flue gas and at high concentrations have the potential to inhibit the growth of microalgae (Yen et al. 2015). At moderate concentrations, however, the nitrogen has the potential to support growth. The negative effects that SOx have on microalgal growth is generally due to a decrease in pH (Yen et al. 2015). This was mitigated in our experiments by including a pH stat system to maintain the pH of the culture between 6.8 and 7.2. The strategy of intermittently sparging the culture with flue gas potentially improved microalgal growth, as was reported by Chen et al. (2012). A comparison of the growth kinetics of SMC-14M at the 1000 L scale demonstrated no significant differences between the flue gas treatments and the pure CO_2_ control. Jin et al. (2020) conducted a comprehensive study using a strain of *Chlorell*a to compare flue gas from a coke oven to air enriched with varying concentrations of CO_2_ and found that the algae actually grew better when aerated with flue gas. Our results demonstrate that SMC-14M can efficiently remove CO_2_ from flue gas. One of the anticipated benefits of recycling CO_2_ in this way is valorization of the biomass produced and recovered.

Two independent growth trials of SMC-14M in 1000L PBRs were conducted specifically to evaluate the biochemical composition of the biomass when cultured on cement plant flue gas emissions. The biomass was harvested at different time points of the batch growth curve to analyze the relationship between growth rate, phase of growth, and the different biomass components such as lipids, carbohydrates, proteins, and minerals. The potential applications of the biomass would partly depend on its biochemical composition. Therefore, the process in use for biomass production including parameters such as culture dilution rate and other growth conditions are important considerations (Dickinson et al. 2013).

It is well known that algal growth rate correlates positively with cellular protein content (Williams and Laurens 2010). Although it is unclear why the biochemical composition of algal biomass changes at different stages of growth, carbohydrates and lipids tend to accumulate to maximum levels under stationary phase when growth rate is low and nutrients become limiting (Zhu et al. 1997). It is not simply the growth rate or phase that determines the biochemical composition. Growth irradiance and temperature impose their own particular constraints on the biochemical composition of the biomass. The specific composition of lipids and amino acids can be altered in response to environmental conditions. In terms of optimizing a system for commercial value, it is necessary to find the point where the maximum quantity of CO_2_ from the emissions is recycled, while also producing the maximum amount of the targeted biomass component. Interestingly, our results demonstrated little impact on lipid, crude protein, and carbohydrate composition throughout growth. This is unusual as there is generally a positive correlation between protein and growth rate which shifts toward energy and carbon storage compounds as growth rate decreases, as has been verified in other studies (Vello et al. 2018). In this study, fertilization of the cultures continued throughout the cultivation period in order to prevent nutrient limitation. Therefore, it was most likely light, and not nutrients, that ultimately limited algal growth in the PBRs aerated with flue gas. The protein and fatty acid content found throughout the culture growth here were relatively invariant and comparable to the results found at stationary phase by Kobayashi et al. (Kobayashi et al. 2013) with *Chlorella sorokiniana*. Some trends in certain biochemical signatures were detected over the course of the cultivation however. For example, n-6 PUFAs (predominantly 16:2n-6 and 18:2n-6) significantly increased, and n-3 PUFAs (solely 18:3n-3) significantly decreased over the cultivation period. This had a significant declining effect (*P*<0.001) on the n-3:n-6 ratio (from 2.9 to 1.7 over 9 days) throughout the production cycle. This same downward n-3:n-6 ratio phenomenon (from 6.3 to 4.6 over 20 days) has been observed for other microalgae strains grown in PBRs (Tibbetts et al. 2020). This finding will have largely different implications depending upon the intended application of the biomass. For instance, any lipid-rich strain containing significant quantities of polyunsaturated fatty acids (PUFAs) with carbon chain lengths of ≥18 would be generally unsuited for biodiesel production (Halim et al. 2012). Conversely, C18 PUFA in both n-3 and n-6 forms may be of higher value for terrestrial animal nutrition, human nutritional supplements, and finfish aquaculture feeds; but high C18 n-6 PUFA (at the expense of n-3 PUFA) is generally undesirable for the high value cold water salmonid and marine fish aquaculture nutrition. This latter point is of particular note here as the SMC-14M biomass in this study is completely devoid of physiologically essential long-chain n-3 PUFA 20:5n-3 (EPA) and 22:6n-3 (DHA), which is typical for the *Chlorella* genus (discussed in (Tibbetts et al. 2017, 2015)).

Research has shown that microalgae are extremely beneficial in environmental protection due to their biosorption capabilities of toxic elements, such as heavy metals, from the environment. Biosorption, the binding of ions to the cell surface, not only allows for favorable metal uptake in terms of environmental protection, but can also allow for the monitoring of metal pollution of a specific area (Attila Kiss 2013). Heavy metals that settle in the environment can bioaccumulate in food chains and can have adverse effects on health with constant exposure. Cement manufacturing can release heavy metal contaminants such as arsenic (As), cadmium (Cd), lead (Pb), mercury (Hg), zinc (Zn), manganese (Mn), selenium (Se), antimony (Sb), cobalt (Co), thallium (Tl), tin (Sn), nickel (Ni), and vanadium (V) into the atmosphere which are then dispersed some distance from the emission source (Arfala et al. 2018). Microalgae act as a major heavy metal sink concentrating the metals in their biomass allowing for a more manageable volume of material (Napan et al. 2015). Trace and heavy metals (HM) were assessed in the biomass and in the supernatant (HM) throughout the growth trial to examine biosorption properties and to evaluate subsequent biomass quality. In the Brite-Box trials, trace metals were added once at the beginning of cultivation as a requirement by microalgae for growth. As expected, our results demonstrate a strong requirement for iron. In terms of heavy metals, there was a trend (albeit not significant) toward greater cadmium, copper, and lead content as the culture aged, and the accumulation of mercury in the biomass throughout the growth trial was significant. Mercury, Cd+ and Pb+ levels in the supernatant, however, were below the detection limit throughout cultivation, which demonstrated the biosorption capabilities of this strain. Species of *Chlorella* are well known to have exceptional capacity for growth as well as physiological and biochemical characteristics which make them useful production strains for many different applications. Nutrition-related applications are of great interest due to their high nutritive significance and high content of bioactive components (Attila Kiss 2013). However, heavy metals in the biomass can greatly restrict the use of this end product for these food/feed applications.

The mercury content of 0.3±0.2 ppm detected initially in the culture is similar to canned tuna, which have standards and are subject to consumption advice. In Canada, a standard maximum limit of 0.05 ppm of mercury in retail fish is enforced by the Canadian Food Inspection Agency (with exceptions for swordfish, shark, etc. which occupy higher trophic levels and have different consumption advice). Similarly, the maximum allowable concentration in animal feed ingredients is 0.1–0.4 ppm (Tibbetts et al. 2015; EU 2006). As cultivation continued, the mercury content increased to 0.9 ±0.7 ppm. High levels of mercury can affect brain function and heart health in humans. If fed to farmed fish in appreciable amounts, the mercury could bioaccumulate to levels making it difficult to justify their use for any kind of food application. Granted, for many cultured fish species, the recommended inclusion levels of chlorophytic microalgae, such as *Chlorella*, in the complete feed are less than 10%; thereby reducing by 10-fold, the final concentrations of these heavy metals contributed to the feed by microalgae. This also demonstrates the importance of evaluating the whole biomass content after integrating carbon capture from alternative sources such as flue gas emissions with microalgal cultivation.

Microalgae have a significant advantage over competing CO_2_ capture technologies like amine scrubbing arising from the variety of value-added products that can potentially be derived from the biomass, including animal feeds, soil conditioners and fertilizers, platform chemicals, and others. However, from purely a cost perspective and with current technologies, microalgae cannot compete head-to-head with these more mature approaches. In the most inexpensive technology for cultivating microalgae—the open raceway pond—CO_2_ fixation costs run approximately $3.22 US/kg of CO_2_ removed, as a global average (Napan et al. (2015)). A recent study estimated the cost of CO_2_ removal by amine scrubbing to be approximately $0.05 US/kg CO_2_ removed, a roughly 64-fold difference (Arfala et al. (2018)). With the relatively high CO_2_ conversion costs associated with algal fixation, the portfolio of products which can potentially be profitably recovered from algal biomass becomes restricted to the “niche” products such as nutraceuticals, nutritional supplements, specialty feeds, or cosmetics. There is a reason to be optimistic that this picture will change however with more focussed R&D on reducing microalgae production and CO_2_ fixation costs. In a recent study, Chiou et al. (2018) conducted sensitivity analysis on biomass production costs and reported a potential reduction of nearly 50% by increasing the solar energy conversion efficiency from the current optimum of 2.7 to 6%, which is highly probable with the application of genetic modification technologies. A further 25% reduction in cost could potentially be gained by reducing the optical path-length in PBRs from 2 to 1 cm (Chiou et al. (2018)). If these improvements could be realized, the costs of CO_2_ fixation by microalgae could be reduced by as much as 75%, resulting in a drop from $ 3.22 US/kg CO_2_ removed to about $ 0.81 US/kg CO_2_ removed, equivalent to a reduction in biomass production cost from $ 5.90 to $ 1.48 US/kg. Reducing algal biomass production and, concomitantly, CO_2_ fixation costs in such a way could potentially expand the reach of algal products into commodities — typically high volume, low-margin products — which are currently inaccessible because of the high costs of production.

In summary, twenty-nine isolates were obtained adjacent to the SNP-MaPP in an attempt to screen for strains with a high potential for converting carbon dioxide from cement kiln flue gas emissions to value-added biomass. Nine strains belonging to the genera *Chlorella* sp. and *Tetradesmus* demonstrated high growth rates, high biomass density, and high continuous productivity in the initial chemostat cultivation screening step. Cultivation of these isolates in bioreactors on-site at the SNP-MaPP demonstrated the ability to successfully convert CO_2_ directly from kiln flue gas emissions into harvestable biomass. One isolate, *Chlorella sorokiniana*, SMC-14M, showed a greater tolerance to flue gas and a higher productivity (carbon conversion) than the others, substantiating the value in strain selection screening. At large scale (1000 L), SMC-14M performed equally well on flue gas as the clean CO_2_ control. The integration of microalgal cultivation and carbon dioxide recycling from emissions gases has a great environmental and economic potential if done properly under a favorable regulatory framework for carbon accounting and emissions costs. The high heavy metal biosorption capacity of the microalgae offers a technology to decrease pollution of the surrounding environment. The biomass itself can be utilized for many applications; however, the biochemical content should be comprehensively examined and controlled to ensure it is appropriate for the intended end use. Without mitigating technologies in place, results from this study demonstrate that the biomass from cement plant flue gas, while nutritious in most respects, may not be safe for certain applications (e.g., human consumption, animal/aquaculture feed, pharmaceuticals, and nutraceuticals) due to marginally high mercury content.

## Data Availability

All data that support the findings of this study are included within this paper. Any additional information will be made available upon reasonable request.
